# A cysteine-rich receptor-like protein kinase CaCKR5 modulates immune response against *Ralstonia solanacearum* infection in pepper

**DOI:** 10.1186/s12870-021-03150-y

**Published:** 2021-08-19

**Authors:** Shaoliang Mou, Qianqian Meng, Feng Gao, Tingting Zhang, Weihong He, Deyi Guan, Shuilin He

**Affiliations:** 1 College of Life Science, Fujian Agriculture and Forestry University, Fujian 350002 Fuzhou, People’s Republic of China; 2 Key Laboratory of Applied Genetics of Universities in Fujian Province, Fujian Agriculture and Forestry University, Fujian 350002 Fuzhou, People’s Republic of China; 3 Key Laboratory of Plant Genetic Improvement, National Education Minister, Comprehensive Utilization Fujian Agriculture and Forestry University, Fujian 350002 Fuzhou, People’s Republic of China; 4 College of Agriculture Science, Fujian Agriculture and Forestry University, Fujian 350002 Fuzhou, People’s Republic of China

**Keywords:** *Capsicum annuum*, CaCRK5, *Ralstonia solanacearum*, Immune response

## Abstract

**Background:**

Cysteine-rich receptor-like kinases (CRKs) represent a large subfamily of receptor-like kinases and play vital roles in diverse physiological processes in regulating plant growth and development.

**Results:**

*CaCRK5* transcripts were induced in pepper upon the infection of *Ralstonia solanacearum* and treatment with salicylic acid. The fusions between CaCRK5 and green fluorescence protein were targeted to the plasma membrane. Suppression of *CaCRK5* via virus-induced gene silencing (VIGS) made pepper plants significantly susceptible to *R. solanacearum* infection, which was accompanied with decreased expression of defense related genes *CaPR1*, *CaSAR8.2*, *CaDEF1* and *CaACO1*. Overexpression of *CaCRK5* increased resistance against *R. solanacearum* in *Nicotiana benthamiana*. Furthermore, electrophoretic mobility shift assay and chromatin immunoprecipitation coupled with quantitative real-time PCR analysis revealed that a homeodomain zipper I protein CaHDZ27 can active the expression of *CaCRK5* through directly binding to its promoter. Yeast two-hybrid and bimolecular fluorescence complementation (BiFC) analyses suggested that CaCRK5 heterodimerized with the homologous member CaCRK6 on the plasma membrane.

**Conclusions:**

Our data revealed that CaCRK5 played a positive role in regulating immune responses against *R. solanacearum* infection in pepper.

**Supplementary Information:**

The online version contains supplementary material available at 10.1186/s12870-021-03150-y.

## Background

When plants are attacked by pathogens, plant immune receptors can detect pathogen infection and elicit a battery of defense responses. As the first line of plant defense, pattern recognition receptors (PRRs) recognize microbe-associated molecular patterns (MAMPs) that are released by the pathogen during infection and trigger plant immunity [1]. An increasing body of evidence suggests that the surface-localized PRRs are either receptor-like kinases (RLKs) or receptor-like proteins (RLPs) in plants [2, 3]. Well-characterized PRRs include the *Arabidopsis* leucine-rich repeat (LRR) receptor kinases FLAGELLIN SENSING 2 (FLS2) and EF-TU RECEPTOR (EFR), which can recognize bacterial flagellin [4] and EF-Tu [5], respectively. RLKs comprise a superfamily of transmembrane proteins, which play a critical role in various signal transduction pathway. In higher plants, there are about 610 RLKs in model plants *Arabidopsis* [6] and near 1000 in rice [7]. A typical RLK contains an extracellular receptor, a transmembrane domain and an intracellular Ser/Thr kinase domain [6]. Although roles for most RLKs are unknown, many RLKs were reported to regulate plant physiological processes, including development, hormone perception and defense responses [8].

Cysteine-rich RLKs (CRKs) are characterized by the presence of one to four copies of Domain of Unknown Function 26 (DUF26) and a C–X8–C–X2–C motif in the extracellular receptor region at the N-terminus [9]. The conserved Cys residues might be required to form the three-dimensional structure of the protein through disulfide bonds [10], can mediate protein-protein interactions [11]. It has been reported that CRKs are involved in the regulation of immune responses [12–14]. Most of the CRKs in *Arabidopsis* are induced by pathogen attack and SA application at the transcriptional level [15, 16]. Some of the CRKs have been functionally characterized in the response to the pathogen infection. For example, CRK45 served as a positive regulator in *Arabidopsis* disease resistance. *CRK45* overexpression enhanced resistance to *Pseudomonas syringae*, whereas the *crk45* mutant was more susceptible to *P. syringae* [16]. LecRK-VI.2, a positive regulator of pattern-triggered immunity (PTI), induces the expression of 7 CRK genes (*CRK4/6/7/13/23/36/37*) in *Arabidopsis*. Among them, overexpression of *CRK4*, *CRK6* and *CRK36* increased disease resistance to the bacterial pathogen *Pst* DC3000 through a positive regulation of PTI response [17]. Moreover, recent studies revealed that some CRKs could form a receptor complex with RLKs and play roles in transducing PTI signals in the plant. It has been reported that increased expression of *CRK28* in *Arabidopsis* enhanced ROS burst and disease resistance to *P. syringae.* CRK28 associated with FLS2/brassinosteroid insensitive 1-associated kinase 1 (FLS2/BAK1) immune complex as well as associated with the closely related CRK29 in *Arabidopsis* [18]. However, it remains unknown how CRKs regulate defense responses in pepper.

Wilt disease is one of the most devastating bacterial diseases in the world that is mainly caused by the soil-borne bacterium *R. solanacearu*m [19, 20]. The pathogen infects a wide range of crops of economic importance, but is particularly devastating to *Solanace*ae plants, such as tomato, tobacco and pepper [21]. Here, we report the identification of a CRK protein, CaCRK5, which functions as a positive regulator in the pepper response to *R. solanacearum* infection. Suppression of *CaCRK5* in pepper plants via virus-induced gene silencing (VIGS) increased susceptibility to the pathogen. In contrast, overexpression of *CaCRK5* in *N. benthamiana* enhanced resistance to pathogen. In addition, electrophoretic mobility shift assay (EMSA) and chromatin immunoprecipitation (ChIP)-qPCR analysis revealed that transcription factor CaHDZ27 up-regulates the expression of *CaCRK5* by binding to its promoter. Furthermore, the data from yeast two-hybrid and bimolecular fluorescence complementation (BiFC) analyses indicate that CaCRK5 interacted with its homolog CaCRK6 on the plasma membrane. These results uncover that CaCRK5 plays a vital role in the regulatory network regulating pepper response to *R.solanacearum* infection, suggesting that CaCRK5 is an effective target for improvement of pepper resistance to wilt disease.

## Results

### CaCRK5 encodes a pathogen induced receptor-like kinase of the arginine aspartate (RD) family

In cDNA-AFLP (cDNA amplified fragment length polymorphism) experiments designed to isolate genes involved in the pepper resistance to the infection of *R. solanacearum*, a partial cDNA fragment of *CaCRK5* was obtained [22]. As its expression was significantly up-regulated after inoculation of *R. solanacearum*, we decided to study the function of this gene further. *CaCRK5* cDNA clones were isolated from a cDNA library made from *R. solanacearum* inoculated leaves of pepper inbred line CM334. The proteins deduced from the cDNA clones of CaCRK5 contained 669 residues. SMART (http://smart.embl-heidelberg.de/) analysis of the domain architecture predicted that CaCKR5 was composed of two cysteine-rich DUF26 domains (PFAM01657, Stress-antifung domain), a transmembrane region and a serine/threonine kinase domain (PFAM07714), and therefore CaCRK5 belongs to the family of cysteine-rich kinases [10]. In addition, CaCRK5 contains a conserved arginine-aspartic (RD) motif in the protein kinase domain (Additional file 1). For most RD kinases, the phosphorylation in the activation loop is crucial for triggering kinase activity which usually displays phosphorylation/autophosphorylation ability. Non-RD kinases usually exhibit lower kinase activities because of the lack of activation loop autophosphorylation. RD and non-RD kinases often cooperate to control innate immune signaling in plants [23–26].

### Genome-wide identification of CRK family in pepper

To gain more information about CRK in pepper, we evaluate the CRK gene family in the pepper genome, Hidden Markov Model (HMM) profile of the DUF26 domain (PFAM01657) was used to search the pepper cultivar CM334 proteins database (http://cab.pepper.snu.ac.kr/). The *Arabidopsis* CRK gene family sequences [10] were also used as query sequences to search against the PGP (Pepper Genome Platform) and the NCBI database. The identified candidates were further subjected to domain analysis using NCBI-CDD and SMART, to ensure the presence of three essential domains for specific CRK proteins including stress-antifung domains, a transmembrane domain, and a kinase domain. A total of 27 CRK genes (CaCRKs) were identified in the pepper cultivar CM334 genome, numbered from CaCRK1 to CaCRK27 according to their localization on chromosomes. Except for CaCRK13/24/25 with only one DUF26 domain, most CRKs contained two DUF26 domains. The amino acid number composing of CaCRK proteins varied from 332 to 1120, and molecular weights from 36.76 kDa to 128.25 kDa. The predicted isoelectric points of CaCRK ranged from 5.53 to 9.37 (Additional file 2).

Chromosome physical localization analysis revealed that 27 CaCRKs were distributed on 7 of the 12 chromosomes in pepper genome. Chromosome 2 contained the largest number of CRKs with 9 genes (34.6 %), whereas chromosomes 12 contained only 1 gene. Gene clustering was the most common feature of the CRK genes distribution. 22 CRKs were identified in tandem repeats on the same chromosome, with no or only one intervening annotated gene. (e.g. CaCRK1-7, CaCRK10/11, CaCRK14/15, CaCRK16-19, CaCRK20/21, CaCRK22/23 and CaCRK24-26). Phylogenetic analysis was performed using the full-length protein sequences of CRK from pepper, and an unrooted phylogenetic tree constructed by the neighbor-joining (NJ) method. The result showed that all CRKs in pepper were classified into four subfamilies designated I to IV according to the phylogenetic relationship. IV is the largest subfamily that contains 13 CRKs, III is the smallest subfamily and comprised of only two CRKs (CaCRK10/11). Subfamily II contains 5 CRKs (CRK13-15 and CRK20/21), and I contains 7 CRKs (CaCRK1-7) which all locate on chromosome 2. CRKs that located in tandem repeats clustered in the same clade in the phylogenetic tree, except CaCRK22/23 (Additional file 3). These results suggested that the expansion of CRKs in pepper might be due to tandem duplication. This distribution and physical clustering pattern are consistent with CRKs in *Arabidopsis *[18] and soybean [27].

### Expression of *CaCRK5* in response to the infection of *R. solanacearum* and treatment with exogenous SA, MeJA and ETH

**Fig. 1:**
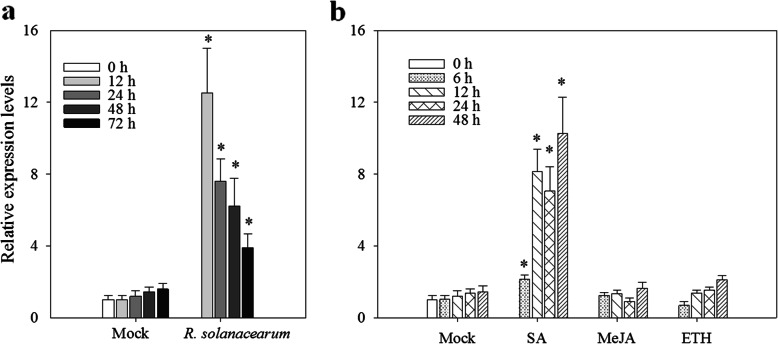
Expression patterns of *CaCRK5* under different treatments. **a** Expression analysis of *CaCRK5* in pepper after inoculation with *R. solanacearum* by qRT-PCR. **b** Expression analysis of *CaCRK5* in pepper responding to SA, MeJA and ETH. The values for mock-treated plants (0 h) were set to a relative expression level at “1”. Error bars represent the mean ± SD of three independent replicates. Asterisks indicate significant differences relative to mock inoculated plants as determined by Mann-Whitney U test (*P* < 0.05)

To characterize the expression pattern of *CaCRK5* in detail, quantitative real-time PCR (qRT-PCR) analyses were performed. 6-week-old pepper plants were leaf-inoculated with *R. solanacearum*. *CaCRK5* was rapidly up-regulated over time after inoculation of *R. solanacearum*, and the highest level of expression was observed at 12 h post inoculation (hpi) with about 12.4-fold of that in control plant (Fig. 1a). Next, we determined the response of *CaCRK5* to the defense-related signaling molecules salicylic acid (SA), methy jasmonate (MeJA) and ethephon (ETH). The results showed that the expression of *CaCRK5* was significantly increased after SA treatment and reached a peak at 48 h. In contrast, indistinguishable change was observed when treated with MeJA or ETH (Fig. 1b). These results suggested that *CaCRK5* was involved in pepper defense against *R. solanacearum* invasion, likely through SA-mediated defense signaling.

### Subcellular localization of CaCRK5

**Fig. 2:**
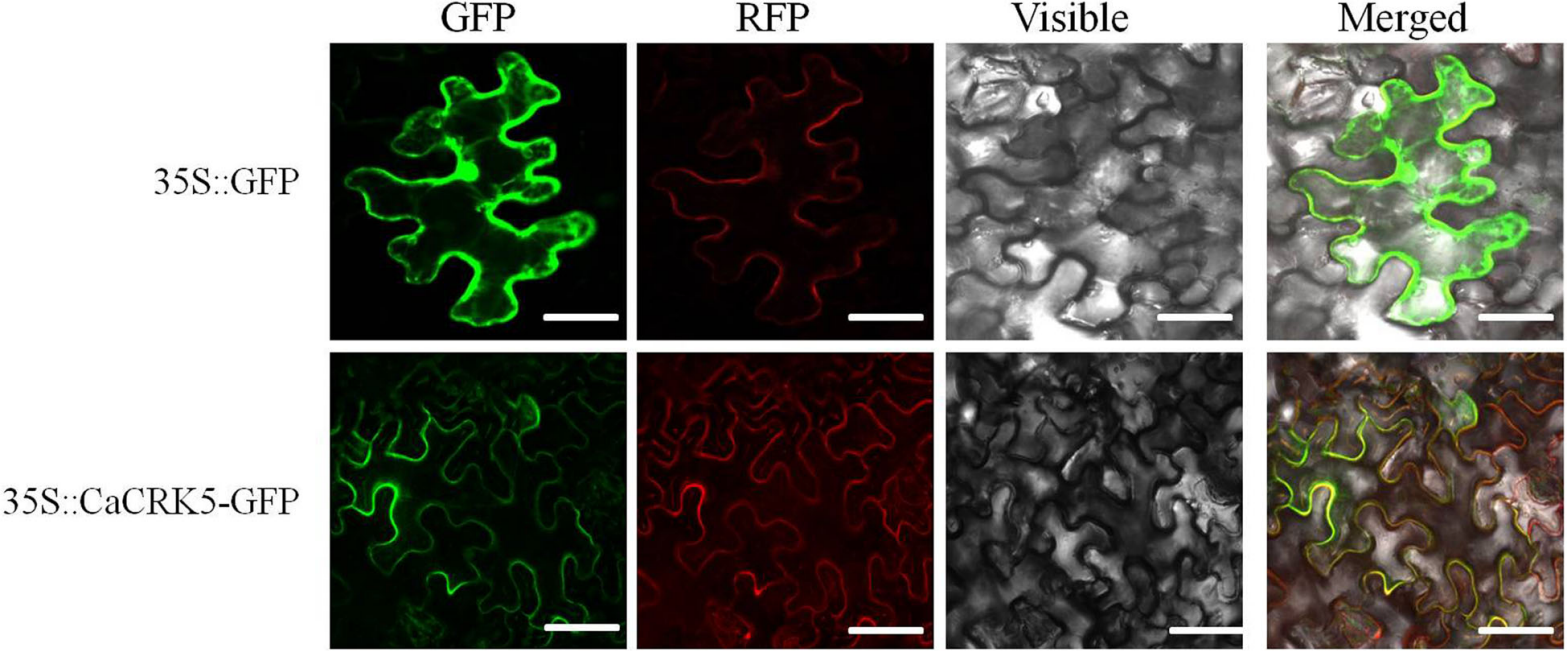
Subcellular localization of CaCRK5. Four-week-old *N. benthamiana* leaves were infiltrated with *Agrobacterium* carrying *35 S*:*GFP*, *35 S*:*CaCRK5-GFP* and *35 S*:*CBL1n-RFP*. Samples were collected 48 h post infiltration. Fluorescence was examined using a confocal microscope. GFP, green fluorescent protein; RFP, red fluorescent protein. Bars, 50 μm

Since CaCRK5 encoded a potential transmembrane domain, it was predicted to localize on the plasma membrane. To test the hypothesis, CaCRK5 was fused with green fluorescent protein (GFP) under the control of the CaMV 35 S promoter. CBL1n protein, which is known localized on the plasma membrane [28], was fused with red fluorescent protein (RFP). *35 S*:*GFP*, *35 S*:*CaCRK5-GFP* were transiently co-expressed with *35 S*:*CBL1n-RFP* in leaf epidermal cells of *N. benthamiana.* As shown in Fig. 2, *35S:GFP* construct served as a negative control, and green fluorescence was ubiquitously distributed throughout the cell. CaCRK5-GFP green fluorescence overlapped very closely with the red fluorescence on the plasma membrane suggesting the plasma membrane localization in the plant cell.

### Silencing of *CaCRK5* in pepper plants increases susceptibility to *R. solanacearum* infection

To assess the role of *CaCRK5* in the interaction between pepper *and R. solanacearum*, loss-of-function experiments in pepper seedlings by virus-induced gene silencing (VIGS) were performed [29]. Specific fragment for *CaCKR5* gene was used to generate VIGS construct, and standard VIGS procedure was performed with pepper phytoene desaturase (CaPDS) construct as the positive control. As shown in Additional file 4, photobleaching was observed in newly emerged true leaves of plants infiltrated with *Agrobacterium* carrying *CaPDS*, indicating that the VIGS system worked efficiently.

**Fig. 3:**
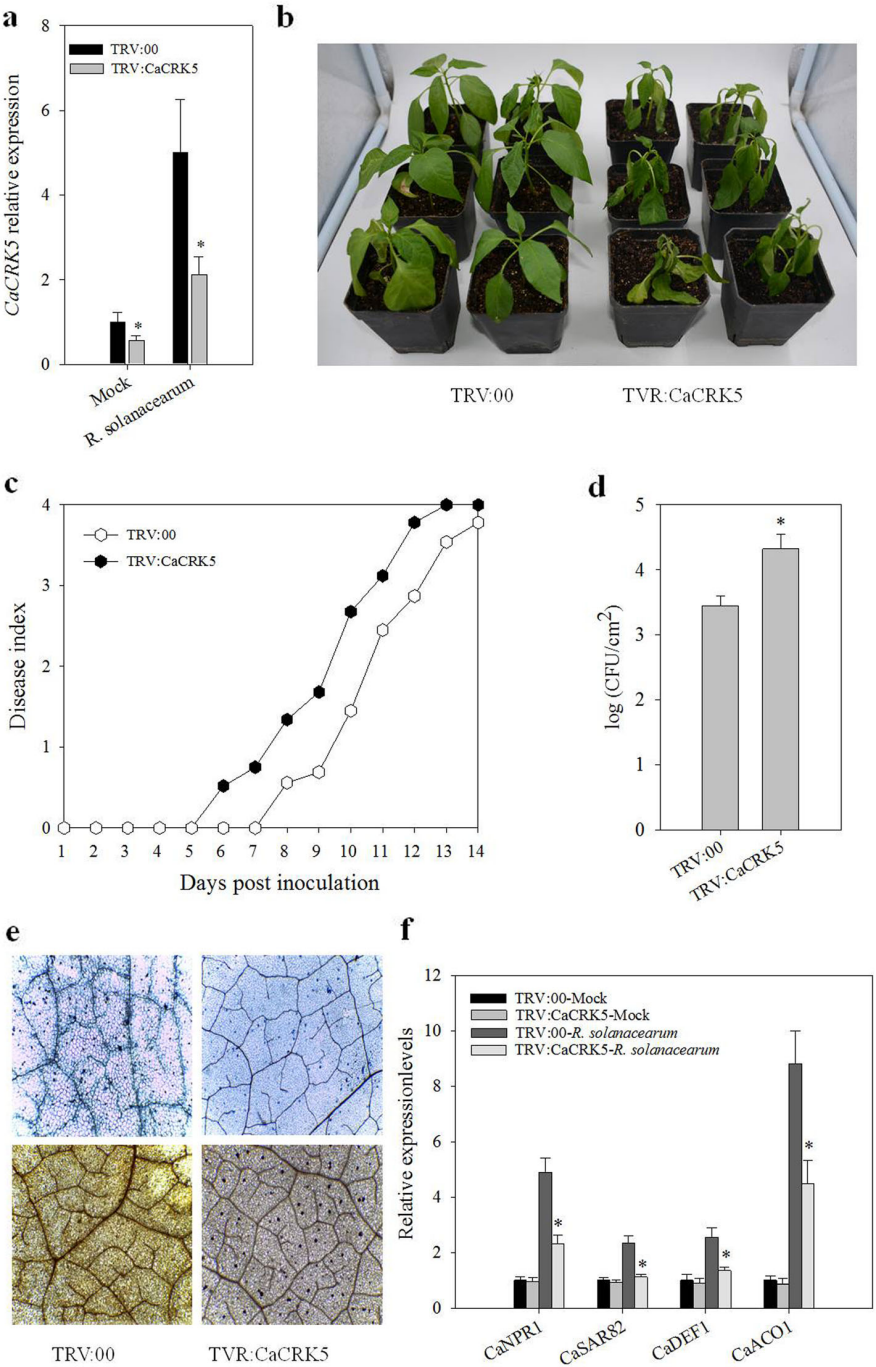
Silencing of *CaCRK5* in pepper enhanced susceptibility to the infection of *R. solanacearum*. **a** qRT-PCR analysis to check the expression of *CaCRK5* in *CaCRK5*-silenced (TRV:CaCRK5) and empty vector control (TRV:00) pepper plants. Error bars represent the mean ± SD of three independent replicates. **b** Phenotypes of control and VIGS pepper plants. After the VIGS was established, 4-week-old plants were root-inoculated with *R. solanacearum*. Photographs were taken at 10 d post *R. solanacearum* infection. **c** Rate of disease index on a scale of 0 to 4 in *CaCRK5*-silenced and control plants. **d** Growth of *R. solanacearum* in leaves of in *CaCRK5* silenced and control plants at 3 d post leaf-inoculation of *R. solanacearum*. The data are shown as means ± SD from three independent repeats (*n* = 3, *, *P* < 0.05, Mann-Whitney U test). **e** Visualization of diaminobenzidine (DAB) and trypan blue staining 48 h post leaf-inoculation with *R. solanacearum*. **f** The expression levels of defense-related genes in *CaCRK5*-silenced and control plants. Asterisks indicate statistically significant differences (Mann-Whitney U test,*P < 0.05)

*CaCRK5* silenced (TRV:CaCRK5) and empty vector control (TRV:00) pepper plants were subjected to *R. solanacearum* challenge. qRT-PCR analysis showed that expression of *CaCRK5* in pepper leaves was significantly down-regulated during *R. solanacearum* infection in VIGS plants (Fig. 3a), indicating that *CaCRK5* were efficiently silenced. Phenotypic analysis indicated that *CaCRK5* silenced pepper plants showed more severe disease symptoms than control plants after *R. solanacearum* infection (Fig. 3b). From 6 days post-inoculation with *R. solanacearum*, *CRK5* silenced pepper plants exhibited a more rapid disease development. The disease index of *CaCRK5* silenced plants was significantly increased, compared to the control plants (Fig. 3c). To address whether silencing of *CaCRK5* affects the growth of *R. solanacearum*, the bacterial population was determined. As shown in Fig. 3d, the growth of *R. solanacearum* was significantly enhanced in *CaCRK5* silenced plants 3 day post leaf-inocualtion, compared with control plants. Based on these observations, we believed that *CaCRK5* participate defense responses in pepper.

Next, we investigated the cell death and oxidative burst in *CaCRK5* silenced and the control leaves. Trypan blue and DAB staining confirmed that hypersensitive cell death and H_2_O_2_ accumulation were significantly reduced in *CaCRK5* silenced leaves 48 h after inoculation with *R. solanacearum* (Fig. 3e), indicating that *CaCRK5* play pivotal roles in early defense response associated with hypersensitive response cell death during *R. solanacearum* infection. We further determined the effects of *CaCRK5* silencing on the expression of defense-related genes in pepper during *R. solanacearum* infection. qRT-PCR analyses showed that *CaCRK5* silencing in pepper leaves significantly attenuated expression of defense-related genes, including *CaNPR1 *[30], *CaSAR8.2 *[31], *CaDEF1 *[32] and *CaACO1 *[33], during *R. solanacearum* infection (Fig. 3f).

### Overexpression of *CaCRK5* in *N. benthamiana* reduces susceptibility to *R. solanacearum* infection

**Fig. 4:**
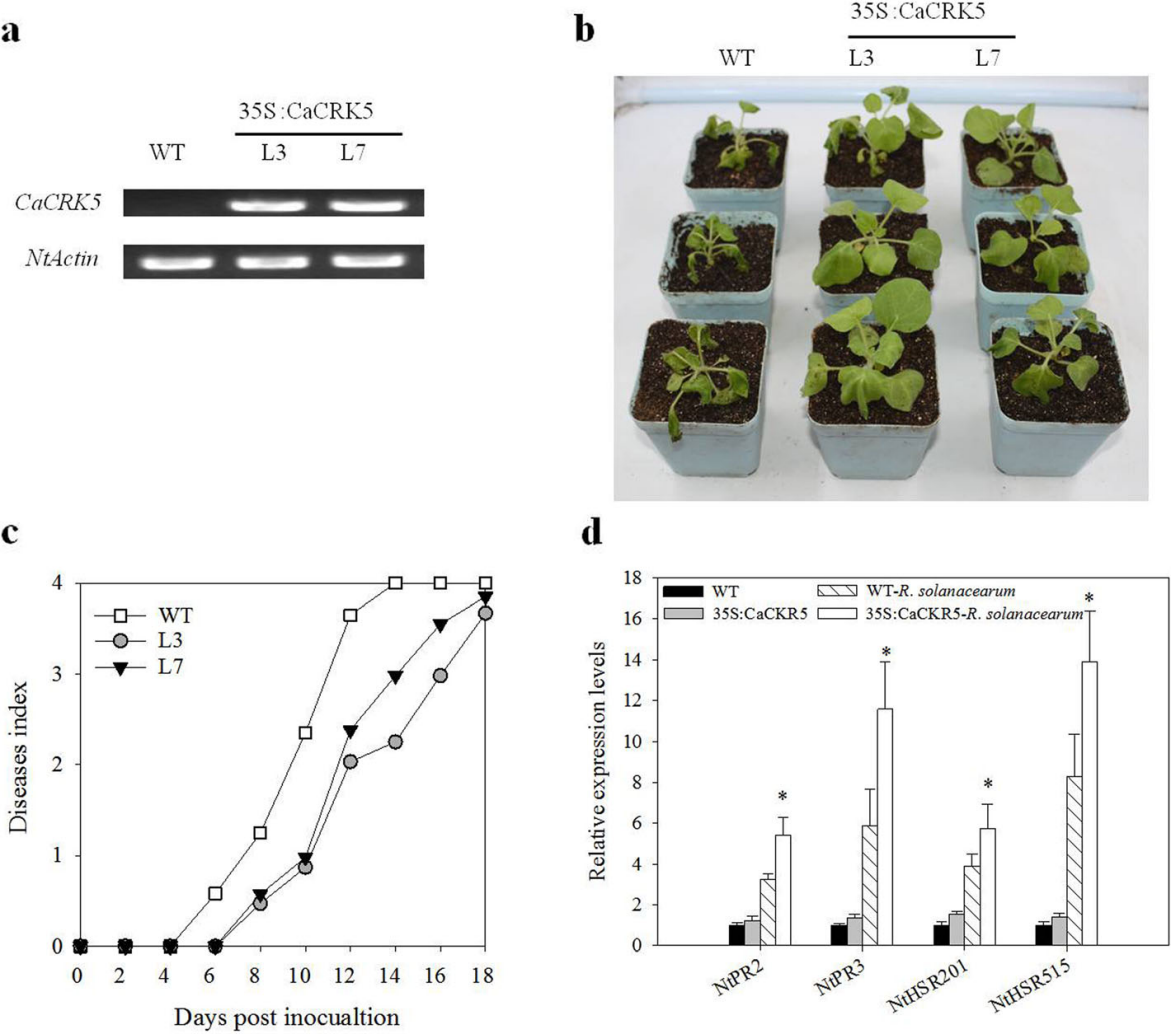
Ectopic expression of *CaCRK5* in transgenic *N. benthamiana* significantly enhances the resistance to *R. solanacearum*. **a** Expression levels of *CaCRK5* in WT (wild-type) and transgenic *N. benthamiana* lines (L3 and L7). **b** Symptoms in WT and overexpressed plants. The photograph was taken 12 d post root-inoculation with *R. solanacearum*. **c** Disease development of bacterial wilt was rated daily on a 0–4 disease index in WT and overexpressed plants. Each data point represents the mean disease index from three independent experiments containing 30 plants in total for each treatment. **d** Expression of four defense-related genes (*NtPR2, NtPR3, NtHSR201* and *NtHSR505*) in in WT and *CaCRK5* overexpressed line L3. Four-week-old WT and transgenic *N. benthamiana* seedlings were leaf-inoculated with *R. solanacearum.* After 48 h, total RNAs were extracted for qRT-PCR assays. Data represent means ± SD (*n* = 3). Asterisks indicate significant differences according to Mann-Whitney U test, *P < 0.05

A gain-of-function approach was also employed to study the function of *CaCRK5* in the defense response. Due to the difficulty in getting regenerated plants for pepper genetic transformation, we choosed *N. benthamiana* plant, which is also a host for *R. solanacearum*. At least 10 transgenic *N. benthamiana* lines were obtained and confirmed by kanamycin resistance analysis. Two T_3_*CaCRK5* overexpressed lines exhibited constitutive expression (Fig. 4a and Additional file 5), and were used in subsequent experiments.

Wild-type and transgenic plants (L3 and L7) at 4 weeks old were root-inoculated with *R. solanacearum*. The effect of *CaCRK5* overexpression on development of bacterial wilt disease was determined. As shown in Fig. 4b, the *CaCRK5* overexpressed plants showed much weaker symptoms at 12 d post-inoculation, compared with the wild-type. The wild-type plants showed wilt symptom from 6 day after inoculation and completely died 14 d after inoculation with *R. solanacearum*. The *CaCRK5* overexpressed plants exhibited a significant delay of wilt symptom compared to wild-type plants (Fig. 4c), showing that overexpression of *CaCRK5* conferred increased disease tolerance to *R. solanacearum* in *N. benthamiana*. In addition, the expression of defense-related genes, including *NtPR2*, *NtPR3*, *NtHSR201 and NtHSR505*, were determined by qRT-PCR. The analysis indicated that expressions of these tested defense-related genes were increased in *CaCRK5* overexpressed plants compared to wild-type plants, during the inoculation of *R. solanacearum* (Fig. 4d). Collectively, these data suggested that *CaCRK5* overexpression enhanced defense responses against *R. solanacearum* infection in *N. benthamiana*.

### The expression of *CaCRK5* is directly regulated by transcription factor CaHDZ27

To better understand the regulatory mechanism of *CaCRK5*, a 2000-bp promoter region upstream of the *CaCRK5* coding sequence was identified. Sequence analysis using the PlantCARE database (http://bioinformatics.psb.ugent.be/webtools/plantcare/html/) suggested that the *cis*-elements in the promoter of *CaCRK5* included two TCA-element involved in salicylic acid responsiveness, five binding sites for MYB transcription factor, and one binding sites for MYC. In addition, a known binding site (CAATTATTG) for the HD-Zip subfamily I member CaHDZ27 was located between positions − 625 and − 617. As CaHDZ27 also regulate the defense in pepper against *R. solanacearum* infection [34], we speculated that *CaCRK5* might be targeted by CaHDZ27.

**Fig. 5:**
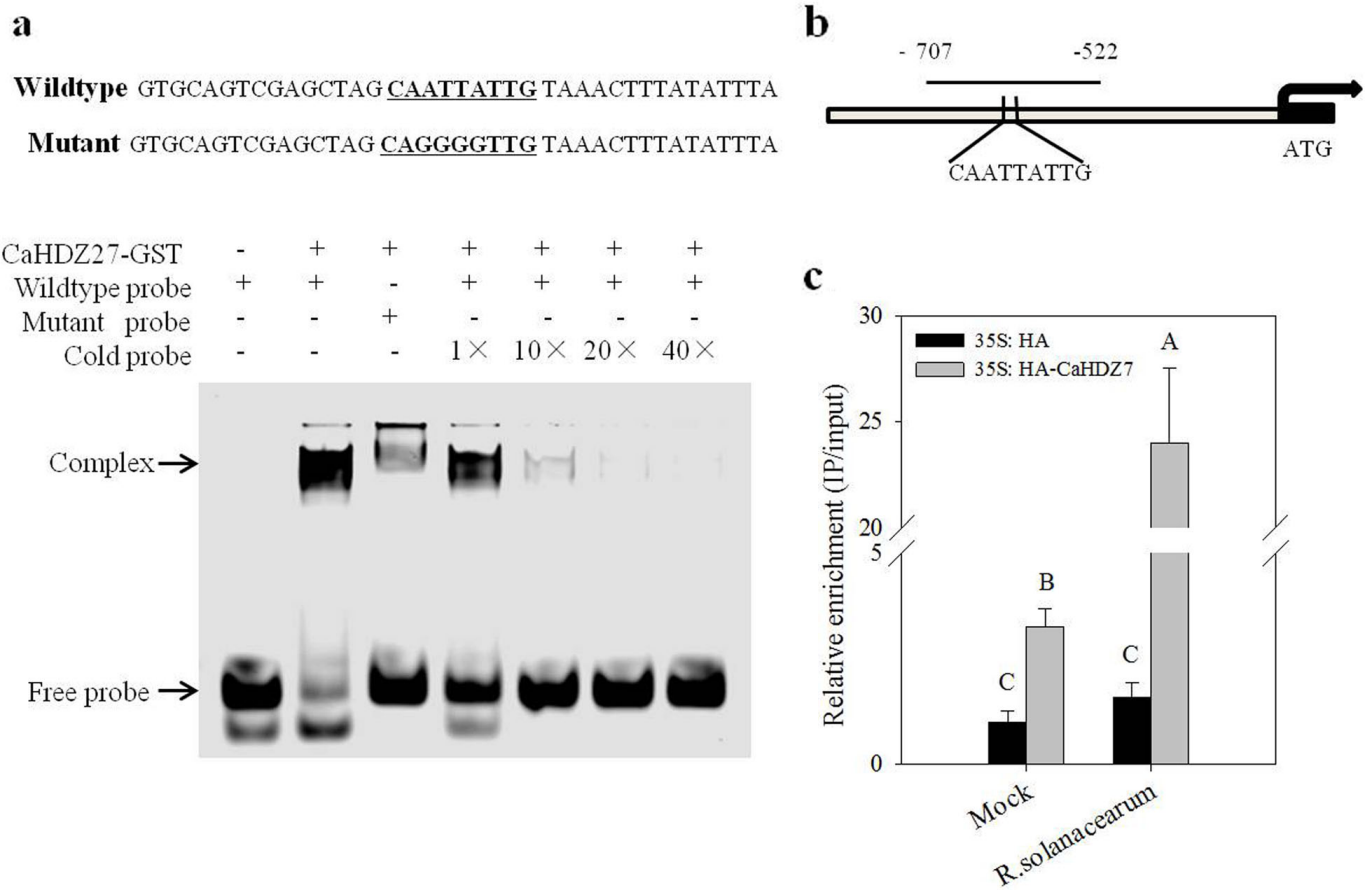
CaHDZ27 binds to the CAATTATTG motif in the promoter of *CaCKR5*. **a** EMSA was used to analyze the interaction of CaHDZ27-GST and a Cy5-labeled probe *in vitro*. The probe sequences containing the CAATTATTG motif in the *CaCKR5* promoter are shown. CaHDZ27-GST purified protein was incubated with Cy5-fluorochrome labeled wild-type probe. Non labeled probes at 10- and 100- fold concentrations were added for the competition test. **b** Schematic representation of the *CaCKR5* promoter used for ChIP-qPCR analysis. The black line indicates the sequence region that was used for ChIP-qPCR. **c**, ChIP-qPCR assay was performed to verify the binding between CaHDZ27 and *CaCKR5* promoter *in vivo*. DNA samples were co-immunoprecipitated with anti-HA antibody. The ChIP value was normalized to its respective input DNA value. Different letters indicate significant differences, as determined by the LSD test (*P* < 0.01)

An electrophoretic mobility shift assay (EMSA) was conducted to access the interaction between CaHDZ27 and the *CaCKR5* promoter, which found CaHDZ27 binds to the Cy5-labeled *CaCKR5* promoter probe. Moreover, binding was gradually attenuated by increasing unlabeled probe concentrations, indicating that CaHDZ27 binds specifically to the CAATTATTG in the *CaCKR5* promoter *in vitro* (Fig. 5a and Additional file 5). Chromatin immunoprecipitation (ChIP)-qPCR was conducted to confirm interaction of CaHDZ27 and the *CaCKR5* promoter *in vivo*. 2 days post infiltration with *Agrobacterium* strain GV3101 containing 35 S:HA-CaHDZ27 or 35 S:HA, the pepper leaves were collected for ChIP assay. Chromatin from these pepper leaves was immunoprecipitated using anti-HA antibodies and enrichment of DNA sample was determined by qRT-PCR. The result showed that CaHDZ27 was significantly enriched in the *CaCKR5* promoter, and the enrichment was significantly enhanced by the inoculation of *R. solanacearum* (Fig. 5b and c), suggesting that CaHDZ27 could bind to the CAATTATTG in the *CaCKR5* promoter *in vitro*, and the binding was enhanced by the *R. solanacearum* infection.

**Fig. 6:**
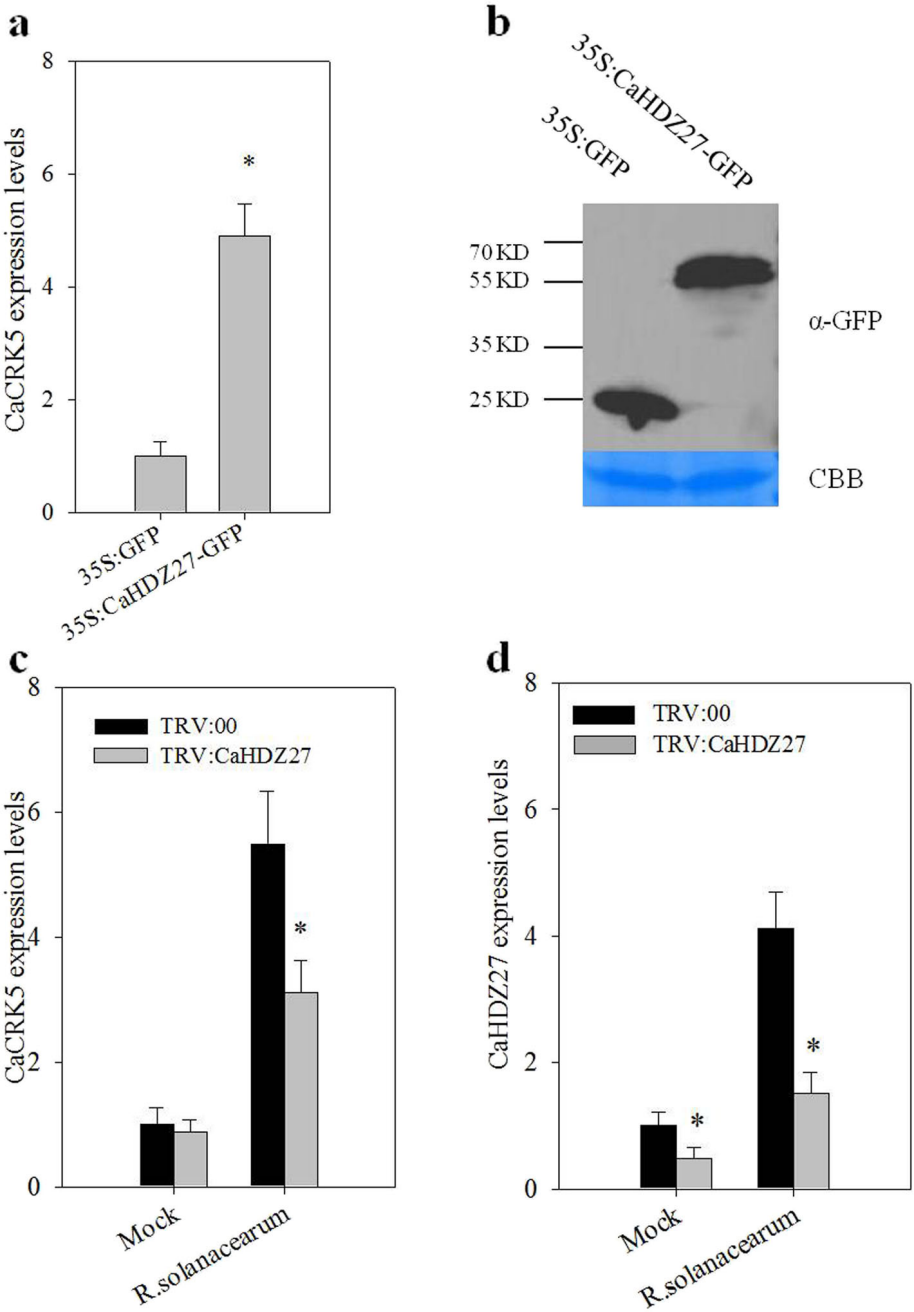
The effect of *CaHDZ27* transient expression or silence on the expression of *CaCKR5.***a** Relative transcript levels of *CaCKR5* in *35 S*:*GFP* or *35 S*:*CaHDZ27-GFP* infiltrated pepper leaves. **b** The presence of CaHDZ27 in pepper leaves identified by immune blot with an anti-GFP antibody 48 h post infiltration. **c-d** Relative transcript levels of *CaCKR5* and *CaHDZ27* in *CaHDZ27* silenced plants by VIGS. Asterisks indicate significant differences at *P* values < 0.05

To further investigate the *CaCKR5* regulation by CaHDZ27 at the transcriptional level, we transiently expressed *35 S*:*CaHDZ27-GFP* in pepper leaves, and 35 S:GFP infiltrated leaves were used as a negative control. qRT-PCR analysis demonstrated that the transcript level of *CaCKR5* was increased in 35 S:CaHDZ27-GFP infiltrated leaves compared with the control (Fig. 6a). The presence of CaHDZ27 in pepper leaves was confirmed by immune blot with an anti-GFP antibody (Fig. 6b and Additional file 5). When *CaHDZ27* was silenced by VIGS, the transcript level of *R. solanacearum*-induced *CaCKR5* is reduced significantly in *CaHDZ27*-silenced plants compared with TRV:00 infiltrated plants (Fig. 6c and d). These data suggested that the transcriptional expression of *CaCKR5* was positively regulated by CaHDZ27.

### CaCRK5 interacts with CaCRK6

Previous studies indicated that plants employ immune receptor complex for sensing MAMPs and effectors to trigger inducible immune defenses [35–38], and a few CRKs have been reported to function in association with each other, such as AtCRK28/AtCRK29 [18] and AtCRK39/AtCRK40 [39]. Based on these reports, we assume that CaCRK5 possibly formed a complex with other homologs including CaCRK6. Thus, we investigated whether CaCRK5 can heterodimerize with the closely related CaCRK6, which has 77.4 % amino acid sequence identity to CaCRK5.

**Fig. 7:**
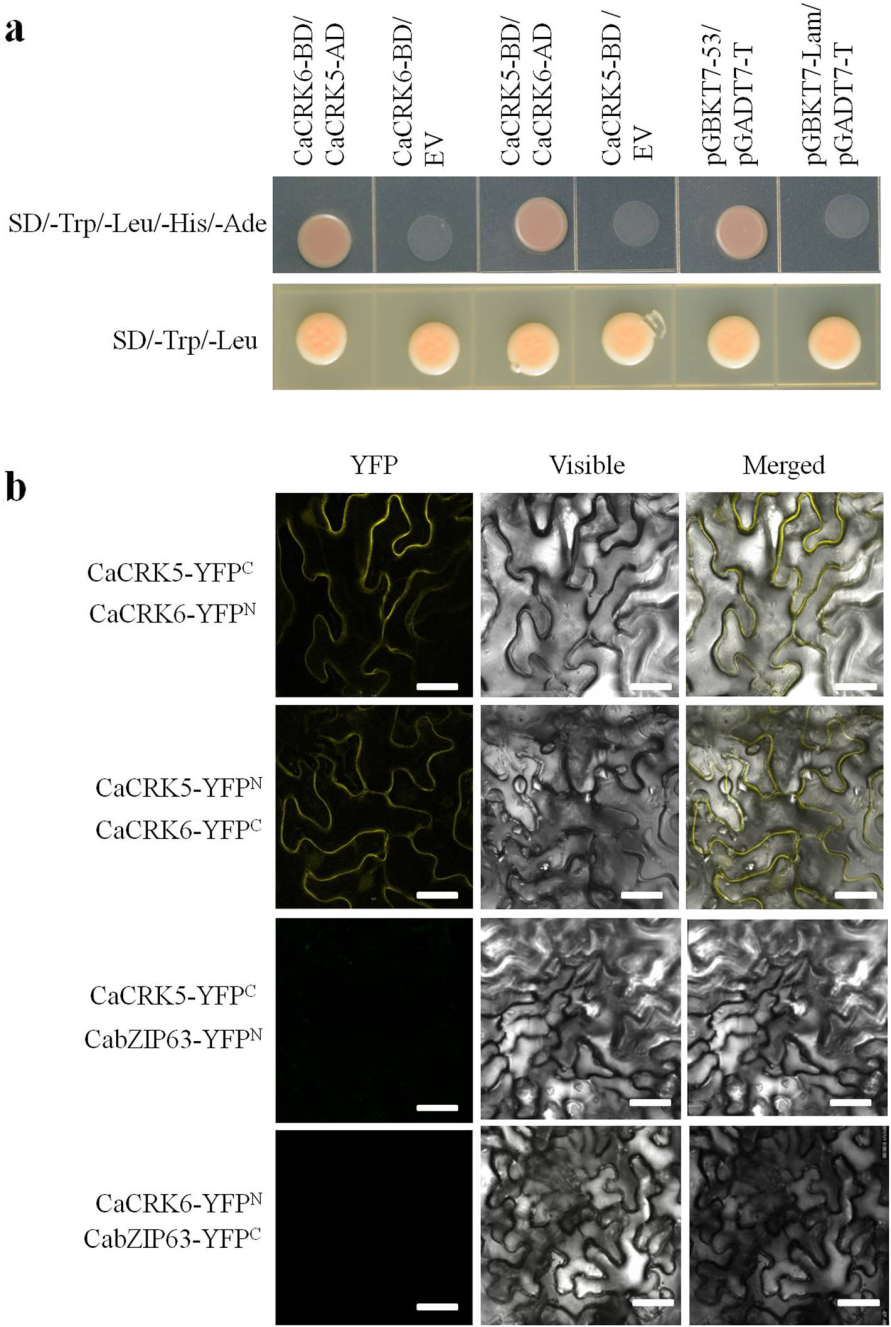
CaCRK5 interacts with CaCRK6. **a** Yeast two-hybrid **(**Y2H) assay. CaCRK5-BD and CaCRK6-AD were co-transformed into ‘Y2H Gold’ yeast cells, which were grown on SD/-Trp-Leu and SD/-Trp-Leu-His-Ade for 3 d. EV indicates the empty vector for pGBKT7. **b** Bimolecular fluorescence complementation (BiFC) assay. YFP fusion constructs were transiently co-expressed in *N. benthamiana* leaves, and BiFC signals were observed under a fluorescence microscope. Bars = 50 μm. The experiment was repeated independently three times with a similar result

The cDNAs of *CaCRK5* and *CaCRK6* were cloned into the pGBKT7 and pGADT7 vectors separately, and generated DNA-binding domain (BD) and activation domain (AD) fusions. BD-CaCRK5/AD-CaCRK6, BD-CaCRK6/AD-CaCRK5 and pGBKT7-53/pGADT7-T (positive control) transformant yeast cells can grow on SD/-Trp-Leu-His-Ade medium. In contrast, no growth was observed in the negative controls. The result indicated that CaCRK5 interacts with CaCRK6 each other in yeast cells (Fig. 7a). To further confirm the interaction between CaCRK5 and CaCRK6, we performed bimolecular fluorescence complementation (BiFC) assays. CaCRK5 and CaCRK6 were fused to the N- and C-terminal ends of yellow fluorescent protein (YFP) to generate CaCRK5-nYFP/CaCRK6-cYFP and CaCRK5-cYFP/CaCRK6-nYFP, respectively. Then, these constructs were transiently co-expressed in *N. benthamiana* leaves. At 48 h post infiltration, the YFP fluorescence signals were determined by confocal microscopy. As showed in Fig. 7b, YFP fluorescence was observed on the plasma membrane in *N. benthamiana* leaves. These results indicate that CaCRK5 heterodimerizes with CaCRK6 on the plasma membrane of plant cells.

## Discussion

### The tandem repeats contributed to the expansion of the CaCRK family in pepper

CRKs are a subfamily of plant RLKs. In this study, 27 CaCRKs were identified in pepper genome; 22 CRKs were present in tandem repeat which made physical clusters at specific positions in the chromosome. Previous studies showed that a large proportion of the RLK genes are found in tandem repeats [40–42]. Tandem repeats tend to be involved in stress response in plants [43–45]. The expansion and degree of tandem repeats are significantly correlated with the stress responsiveness of RLK genes [43]. The tandem repeats of CaCRKs in pepper may facilitate the stress adaptive evolution.

### *CaCRK5* played a positive role in the pepper resistance against *R. solanacearum*

Previous studies have shown that some CRK members including AtCRK29 in *Arabidopsis* were induced in response to treatment with pathogens, SA and flg22 [18, 46, 47], suggesting a role for these CRKs in plant immunity. The CaCRK5 closest homolog in *Arabidopsis* is AtCRK29 protein with 41.6 % identity to CaCRK5. In the study, expressions of *CaCRK5* were increased over time after inoculation of *R. solanacearum*, with high levels of gene expression at the early stage of infection. *CaCRK5* was strongly up-regulated after treatment with SA, and no significant change of *CRK5* was observed treated with MeJA and ETH. Signaling mediated by SA, JA or ET is involved in plant defense response to different pathogens [48–50]. SA generally acts in positively in plant response to biotrophic pathogen [51], while JA and ET are usually regarded as the primary regulators during plant response to necrotrophic pathogens [52]. As *CaCRK5* was induced by the infection of *R. solanacearum* and SA, and not by MeJA and ETH, we speculate that *CaCRK5* acts as positive regulator during the early period of pepper response to *R. solanacearum*, when *R. solanacearum* is on its biotrophic stage.

Based on the subcellular localizations of GFP fusions, CaCRK5 is very likely localized on the plasma membrane. Several other CRKs also reported to localize on the plasma membrane, including CRK4, CRK6, and CRK36 in *Arabidopsis* [17] and GbCRK18 in cotton [53]. The TRV-based VIGS system was used to investigate the function of *CaCRK5* in the response to *R. solanacearum* infection. Expression of *CaCRK5* was significantly decreased in *CaCRK5*-silenced pepper plants during *R. solanacearum* infection. *CaCRK5*-silenced pepper plants resulted in an increased susceptibility to *R. solanacearum*, and pepper leaves significantly compromised H_2_O_2_ accumulation and cell death. These findings suggested that *CaCRK5* may function as a positive regulator in pepper resistance against *R. solanacearum* infection. Consistently, *CaCRK5* overexpression *N. benthamiana* plants exhibited enhanced resistance against infection by *R. solanacearum*.

### *CaCRK5* was transcriptionally regulated directly by CaHDZ27

The HD-Zip transcription factors consist of four subfamilies (HD-Zip I–IV) based on their sequence similarities [54], and HD-Zip I was reported to play roles in biotic and abiotic stress responses [55]. CaHDZ27, a HD-Zip I member, acts a positive regulator in pepper response to *R. solanacearum* infection [34]. Here we showed that CaHDZ27 acts as a positive regulator of *CaCRK5*, CaHDZ27 binds to a 9-bp CAATTATTG motif of the *CaCRK5* promoter revealed by EMSA and ChIP-qPCR. Expression of CaCRK5 is increased in CaHDZ27 transiently expressed pepper leaves, and decreased in *CaHDZ27*-silenced pepper plants with the inoculation of *R. solanacearum*. Moreover, CaCRK5 play a positive role in pepper resistance against *R. solanacearum* infection, consistent with the role of CaHDZ27. These results suggest that CaCRK5 and CaHDZ27 coordinate to regulate the pepper defense against *R. solanacearum* infection.

### CaCRK5 heterodimerizes with CaCRK6

CRK can form heterodimerization with closely related homologs [18, 39]. Yeast two-hybrid and bimolecular fluorescence complementation analyses indicated that CaCRK5 heterodimerizes with CaCRK6 in vivo in both yeast and plant cells. Recent evidence suggests that pattern recognition receptors (PRRs) enable the rapid recruitment of other RLKs, including CRKs, to enhance PRR signaling intensity [18, 46]. CaCRK5 might act in concert with CaCRK6 and other RLKs in the pepper defense against *R. solanacearum* infection.

## Conclusions

In summary, our data suggest that *CaCRK5* significantly contributes to immune defense against *R. solanacearum* in pepper and *N. benthamiana*. VIGS in pepper plants and gain-of-function analyses of *CaCRK5* in *N. benthamiana* revealed that *CaCRK5* positively regulates plant immune responses. In addition, CaHDZ27 promotes the expression of *CaCRK5* by directly binding to its promoter. We propose that the pepper defense response mediated by CaCRK5 might be enhanced by CaHDZ27. CaCRK5 is involved in the pepper innate immune response and may be promising target for genetic engineering to enhance pepper resistance against wilt disease.

## Methods

### Plant materials and pathogen inoculation

Pepper (*Capsicum annuum* cultivar L. cv. Fj8) was obtained from the pepper breeding group at Fujian Agriculture and Forestry University (FAFU), which exhibited medium-resistance to *R. solanacearum* infection identified by Prof. Shuilin He at FAFU in China. Pepper and *N. benthamiana* plants were grown in plastic pots containing steam-sterilized soil at 26 °C with a long-day photoperiod (16 h light/8 h dark) with 60 % relative humidity.

Virulent strains FJC100301 of *R. solanacearum* was used in this study. *R. solanacearum* strain was grown on casamino peptone agar (CPG) plates containing triphenyltetrazolium chloride (TZC), and a single colony was cultured in PSA (potato saccharose agar) medium at 28 °C for 2 days. Bacteria cell solution was harvested with sterile 10 mM MgCl_2_, and the suspension was adjusted to 1.0 × 10^8^ CFU/mL. Inoculation of bacteria was carried out by root irrigation. After cutting the plant roots, 30 mL bacterial solution was introduced into the soil in each pot. To inoculate plant leaves, 10 µL of the suspension of *R. solanacearum* was infiltrated into the leaf of 6-week-old pepper plant using a syringe without a needle, and sterilized MgCl_2_ solution was infiltrated in mock-treated plants. For each plant, a disease index on a scale of 0 to 4 was calculated as described previously [22].

### Bacterial strain growth

The growth of *R. solanacearum* was measured by quantifying bacterial multiplication in pepper leaves. To do this, four leaf discs of 0.5 cm diameter were collected per samples at 3 days post inoculation, and were ground in 1 mL of sterile water. Diluent was plated on TZC solid medium and was incubated at 28 °C for 48 h. Colonies were counted to determine as CFUs per square centimeter of leaf tissue. Pathogen growth experiment was repeated three times, and contained at least three plants per genotype.

### Subcellular localization

For the subcellular localization assay, cDNA was used as a template to amplify CaCRK5 coding region (5-ATGCCTATTCAGAAGTGGC-3 and 5-TCATGCCTGAATACGTGATG-3), then cloned into pMDC83 vector resulting in 35 S:CaCRK5-GFP construct, which was introduced into *Agrobacterium tumefaciens* strain GV3101 by freeze-thaw method. *A. tumefaciens* carrying 35 S:CaCRK5-GFP/35S:CBL1n-RFP (plasma membrane marker) or 35 S:GFP/35S:CBL1n-RFP (1:1 ratio; OD600 = 0.8) were resuspended in the buffer [10 mM 2-(N-morpholino)-ethanesulfonic acid, 10 mM MgCl_2_ and 200 µM acetosyringone, pH5.7], and infiltrated in the leaves of *N. benthamiana* (4-week-old) using the syringe [22]. *A. tumefaciens* infiltrated *N. benthamiana* plants were grown at 26 °C with a 16 h light/8 h dark cycle. For each construct combination, three seedlings of *N. benthamiana* were used, and three leaves per seedling were infiltrated with *A. tumefaciens* cultures. Fluorescence of GFP and RFP in the transformed leaves was imaged using a confocal laser scanning microscope (TCSSP8; Leica, Solms, Germany).

### Virus-Induced Gene Silencing

The tobacco rattle virus (TRV)-based virus-induced gene silencing system was used for gene silencing in pepper. To achieve *CaCRK5* or *CaHDZ27* specific silencing, the DNA fragments in 3’-untranslated region of *CaCRK5* (5-ACTATTCTCACAGCGAC-3 and 5-GAATTCGAACAAATAACA-3) and *CaHDZ27* (5-TTCCACAAGAGAATAGTG-3 and 5-GGAACAAAGCTAATAAA-3) were PCR amplified, then cloned into pTRV2 vector containing part of the tobacco rattle virus (TRV) genome to generate TRV2:CaCRK5 or TRV2:CaHDZ27. These plasmids were introduced into *A. tumefaciens* strain GV3101 by freeze-thaw method. The Phytoene Desaturase (PDS) is used as an indicator gene, its expression is reduced sufficiently to yield a photobleached phenotype. *A. tumefaciens* GV3101 carrying TRV1 with pTRV2:00 (empty vector) or TRV2:CaCRK5 or TRV2:CaHDZ27 was co-infiltrated into the fully expanded cotyledons of pepper plants. Then, the plants were incubated at 16 °C for 56 h, and grown at 26 °C. The experiments were performed with at least 50 plants per treatment and repeated three times.

### Exogenous hormones treatments

4-week-old pepper plants were used to analyze the expression of *CaCRK5* in response to exogenous application of hormones. Plants were sprayed with solutions of 1 mM SA (in 10 % distilled ethanol), 100 µM MeJA (in 10 % distilled ethanol) and 100 µM ETH (in sterile double-distilled H_2_O), respectively. Mock plants were sprayed with corresponding solvent or ddH_2_O.

### RNA extraction and quantitative real-time RT-PCR

Leaves from three different pepper or *N. benthamiana* plants were harvested, and total RNA was extracted using TRIzol Reagent (Invitrogen, Carlsbad, CA, USA). RNA concentration was determined by Nanodrop 2000 (Thermo Fisher Scientific), and the integrity was examined using agarose gel electrophoresis. Reverse transcription was performed using HiScript® Q RT SurperMix for qPCR reagent Kit with gDNA wiper according to the manufacturer’s instructions (Vazyme), and a non-template negative control was used to detect the primer dimerization. The real-time RT-PCR analysis was performed using a Bio-Rad real-time PCR system (Foster City, CA, USA.) and the SYBR Premix ExTaq II system (Takara). The relative expression of the *CaCRK5* and defense-related genes were calculated with the 2^−ΔΔCt^ method. To characterize the expression of the defense-related genes (*CaNPR1*, *CaSAR8.2*, *CaDEF1* and *CaACO1*) in *CaCRK5* silenced pepper, the relative transcript levels were normalized to the control (TRV:00) using the *CaActin* and *Ca18S rRNA* genes as internal references. To assess the expression of the defense defense-related genes (*NtPR2*, *NtPR3*, *NtHSR201 and NtHSR505*) in *CaCRK5* transgenic *N. benthamiana* plants, the relative transcript levels were normalized to the wild type using the *NtActin* and *NtEF1α* genes as internal references. The primers were listed in Additional file 6, and melting curves were listed in Additional file 7.

### Yeast two-hybrid analysis

The pGADT7 vector was used for GAL4 AD, and the pGBKT7 vector was used for GAL4 BD. ORFs of CaCRK5 and CaCRK6 were amplified and cloned into pGADT7 to generate constructs designated AD-CaCRK5 and AD-CaCRK6. The cDNAs of CaCRK5 and CaCRK6 were amplified and cloned into pGBKT7 to generate constructs designated BD-CaCRK5 and BD-CaCRK6. The corresponding AD and BD plasmids were co-transformed into Y2H Gold yeast strain. Transformants containing the indicated plasmids were spotted onto the SD (Synthetic Dextrose) screening medium, and incubated at 30 °C till the formation of colonies. After selection on SD/-Trp-Leu medium, yeast cells were transferred to SD/-Trp-Leu-His-Ade medium for evaluation of interaction.

### Bimolecular fluorescence complementation (BiFC) assay

To generate BiFC constructs, CaCRK5 and CaCRK6 full length cDNAs were cloned into puc-SPYNE^GW^ (CaCRK5-YFP^N^ and CaCRK6-YFP^N^) and puc-SPYCE^GW^ (CaCRK5-YFP^C^ and CaCRK6-YFP^C^) vectors, which contain the N-terminal or C-terminal fragment of YFP (nYFP or cYFP, respectively). Since CabZIP63 acts as a transcription factor, CabZIP63-YFP^N^ and CabZIP63-YFP^C^ were used as the negative control of CaCRK6-YFP^N^ and CaCRK6-YFP^C^, respectively. These constructs were introduced into *A. tumefaciens* strain GV3101 by freeze-thaw method. For transient expression, cells mixture of *A. tumefaciens* strain GV3101 carrying each construct was used to infiltrate leaves of 4-week-old *N. benthamiana* plants. For microscopic analysis, lower epidermis cells were observed using a confocal laser scanning microscope.

### *N. benthamiana* transformation

Transgenic *N. benthamiana* plants were generated by using *A. tumefaciens* mediated *N. benthamiana* leaf disc transformation [56]. The CaCRK5 coding region was cloned into pK7WG2 vector to get *35 S*:*CaCRK5* construct, then it was introduced into *A. tumefaciens* GV3101 and used to transform *N. benthamiana*. Fresh leaf discs were cut from *N.benthamiana*, and immersed in the GV3101 cell suspension (OD = 0.6) for 7–10 min. After drying on sterile paper, leaf discs were transferred to basal Murashige and Skoog (MS) agar medium and incubated at 28 °C for 2–3 days in the dark. After cleaning with sterile water, leaf discs were placed on the selection medium containing kanamycin. At least ten independent *N. benthamiana* transgenic lines were obtained, and checked the expression of *CaCRK5* by RT-PCR. The selected transgenic lines were self-pollinated, T_3_ seeds were obtained and planted on MS agar plates containing 50 µg of kanamycin per milliliter for use in the study.

### Histochemical staining

Histochemical staining was performed as previously described [22]. The infiltrated leaves were stained with trypan blue to visualize the cell death response and DAB solution for H_2_O_2_ detection.

### Electrophoretic Mobility Shift Assay (EMSA)

Expression of the CaHDZ27-GST fusion protein was induced in transformed *E. coli* BL21 cells, by adding IPTG (isopropyl β-D-1-thiogalactopyranoside, 0.05 mM) at 37 °C for 4 h. The recombinant proteins were extracted from the cells and purified using BeaverBeads™ GSH protein purified kit according to manufacturer’s instructions.

The EMSA was performed as described previously [57]. Cy5-labelled DNA fragments were synthesized and used as probes, while unlabeled DNA of the same sequence was used as a competitor.

### Chromatin immunoprecipitation (ChIP)-qPCR analysis

ChIP assay was performed using the method as described previously [22]. Chromatin was extracted from pepper leaves 2 days post infiltration with 35 S:HA-CaHDZ27 and cross-linked with 1 % formaldehyde (v/v). The chromatin was broken into fragments by probe sonicator to an average length of 300 to 500 bps. The fragmented DNA was immunoprecipitated with anti-HA antibody. Enrichment of DNA samples was analyzed by quantitative real-time PCR. The values are presented as the relative enrichment ratio, and the value for the negative control (35 S:HA, mock treatment) was set to “1”. Primers used for ChIP-qPCR are listed in Additional file 6.

### Bioinformatic tools

Amino acid sequences of CRKs in pepper were extracted from CM334 (v1.55) proteins database (http://cab.pepper.snu.ac.kr/). Structural domains of CKRs were analyzed using NCBI-CDD (https://www.ncbi.nlm.nih.gov/Structure/cdd/wrpsb.cgi) [58] and SMART (http://smart.embl-heidelberg.de/) [59]. Alignment of the deduced kinase domain of CaCRK5 with AtCRK28 and AtBAK1 was carried out using DNAMAN 6.0 software [60]. The function prediction of *cis*-regulatory elements within promoter of CaCRK5 was performed using PlantCARE [61].

Essential information of all CKRs in pepper including molecular weight and the theoretical pI were evaluated using the ProtParam tool (http://web.expasy.org/protparam/). An unrooted phylogenetic tree was built by MEGA version 7.0 software [62], using neighbor-joining method with 1000 bootstrap trials. The chromosomal localization of CRK gene family members in pepper were verified from the CM334 (v1.55) proteins database, chromosomal images were drawn using MapInspect 1.0 software (https://mapinspect.software.informer.com/).

## Supplementary Information

**Additional file 1. ** Schematic diagram of CaCRK5 protein domain architecture. It contains the signal peptide (SP), two domains 26 of unknown function (DUF26), transmembrane domain (TM) and kinase domain.

**Additional file 2. ** Essential information of CRKs gene family in pepper.

**Additional file 3. ** Chromosomal location and phylogenetic analysis of CRK gene family. a Physical genome distribution of*CRK* gene family in pepper. b Phylogenetic tree was generated based on the amino acid sequences of *CRK* genes in pepper using the neighbor-joining method with 1000 bootstrap replicates in MEGA version 7.0.

**Additional file 4. ** Photobleaching phenotype of peppers infiltrated with TRV:CaPDS for 15 d.

**Additional file 5.** Original images for Fig. 4a, Fig. 5a and Fig. 6b.

**Additional file 6. ** The information of realt time PCR primers.

**Additional file 7. ** Melting curves in real time PCR.

## Data Availability

The sequencing data described in this study are available in pepper genome database (http://cab.pepper.snu.ac.kr/) under the accession numbers listed in Additional file 2. The datasets generated and/or analysed during the current study are included in this published article and its additional files. Any reasonable requests will be available from the corresponding author.

## Abbreviations

CRK: Cysteine-rich receptor-like kinase
VIGS: Virus-induced gene silencing
PRR: Pattern recognition receptor
MAMP: Microbe-associated molecular pattern
PTI: Pattern-triggered immunity
HMM: Hidden Markov Model
*R. solanacearum*: *Ralstonia solanacearum*
*N. benthamiana*: *Nicotiana benthamiana*
hpi: Hours post inoculation
SA: Salicylic acid
MeJA: Methy jasmonate
ETH: Ethephon
CFU: Colony-forming unit
DUF26: Domain of Unknown Function 26
RLK: Receptor-like kinases
RLP: Receptor-like proteins
LRR: Leucine-rich repeat
FLS2: FLAGELLIN SENSING 2
EFR: EF-TU RECEPTOR
EMSA: Electrophoretic mobility shift assay
ChIP: Chromatin immunoprecipitation
BiFC: Bimolecular fluorescence complementation
cDNA-AFLP: cDNA amplified fragment length polymorphism
PGP: Pepper Genome Platform
