# Supplementary figures and images for: A cysteine-rich receptor-like protein kinase CaCKR5 modulates immune response against Ralstonia solanacearum infection in pepper
Source: BMC Plant Biol. 2021 Aug 19;21:382. doi: 10.1186/s12870-021-03150-y (PMC8375189; doi:10.1186/s12870-021-03150-y)

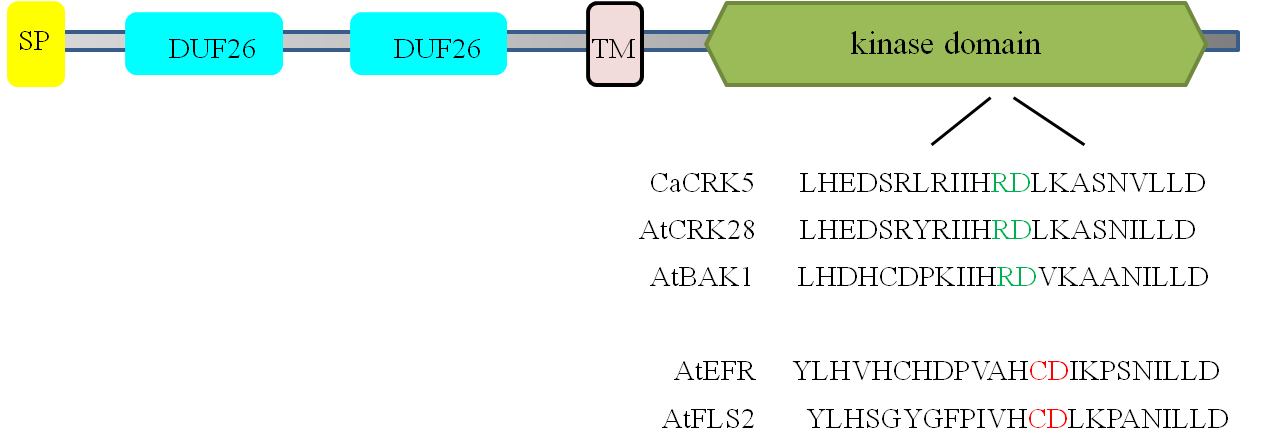

Supplement: Supplementary file 1 — Additional file 1. Schematic diagram of CaCRK5 protein domain architecture. It contains the signal peptide (SP), two domains 26 of unknown function (DUF26), transmembrane domain (TM) and kinase domain. [file 12870_2021_3150_MOESM1_ESM.jpg]

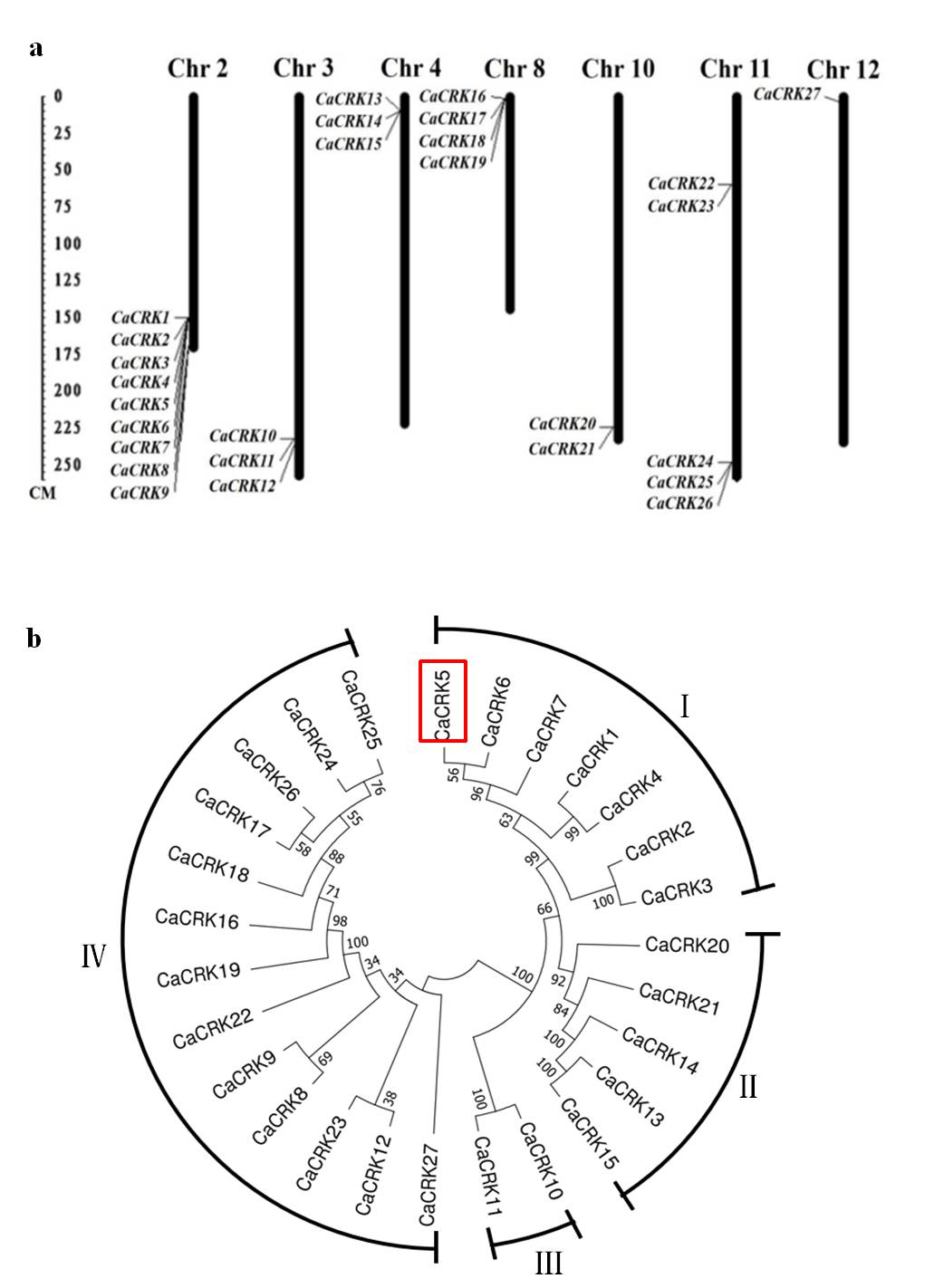

Supplement: Supplementary file 3 — Additional file 3. Chromosomal location and phylogenetic analysis of CRK gene family. a Physical genome distribution ofCRK gene family in pepper. b Phylogenetic tree was generated based on the amino acid sequences of CRK genes in pepper using the neighbor-joining method with 1000 bootstrap replicates in MEGA version 7.0. [file 12870_2021_3150_MOESM3_ESM.jpg]

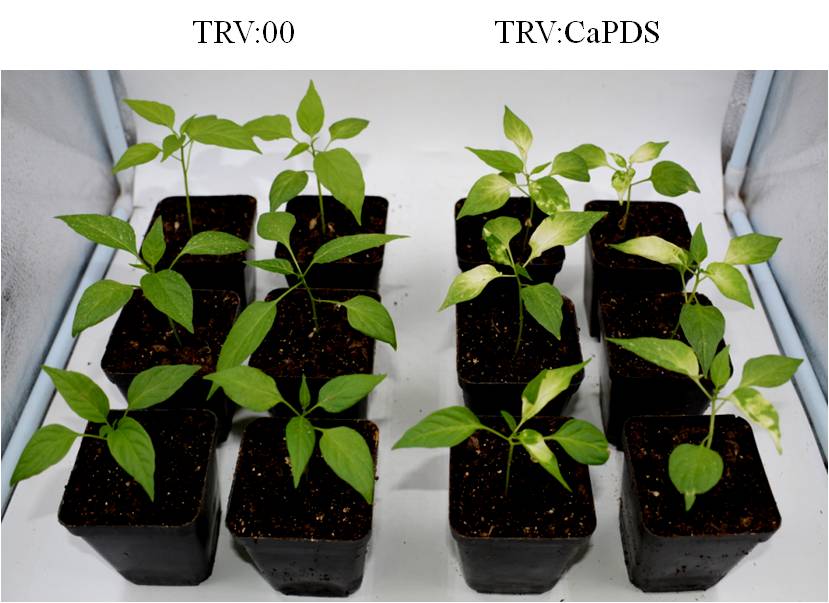

Supplement: Supplementary file 4 — Additional file 4. Photobleaching phenotype of peppers infiltrated with TRV:CaPDS for 15 d. [file 12870_2021_3150_MOESM4_ESM.jpg]
