# Supplementary material for: A cysteine-rich receptor-like protein kinase CaCKR5 modulates immune response against Ralstonia solanacearum infection in pepper
Source: BMC Plant Biol. 2021 Aug 19;21:382. doi: 10.1186/s12870-021-03150-y (PMC8375189; doi:10.1186/s12870-021-03150-y)
Supplement: Supplementary file 2 — Additional file 2. Essential information of CRKs gene family in pepper. [file 12870_2021_3150_MOESM2_ESM.docx]

**Additional file 2.** Essential information of CRKs gene family in pepper

| **Gene name** | **Genome accession*** | **Length of amino acid sequence/aa** | **Molecular weight/Kda** | **The theoretical pI** |
| --- | --- | --- | --- | --- |
| *CaCRK1* | CA02g17760 | 661 | 73.670 | 8.330 |
| *CaCRK2* | CA02g17770 | 653 | 72.95 | 6.42 |
| *CaCRK3* | CA02g17780 | 631 | 70.62 | 6.65 |
| *CaCRK4* | CA02g17810 | 709 | 79.36 | 8.58 |
| *CaCRK5* | CA02g17820 | 669 | 74.41 | 6.58 |
| *CaCRK6* | CA02g17830 | 677 | 75.70 | 7.14 |
| *CaCRK7* | CA02g17850 | 530 | 59.81 | 8.35 |
| *CaCRK8* | CA02g29840 | 680 | 75.12 | 7.93 |
| *CaCRK9* | CA02g29870 | 661 | 72.93 | 8.72 |
| *CaCRK10* | CA03g25110 | 687 | 76.80 | 8.41 |
| *CaCRK11* | CA03g25130 | 577 | 63.75 | 5.81 |
| *CaCRK12* | CA03g26520 | 675 | 75.09 | 6.89 |
| *CaCRK13* | CA04g03070 | 592 | 65.94 | 5.96 |
| *CaCRK14* | CA04g03300 | 670 | 74.48 | 5.53 |
| *CaCRK15* | CA04g03310 | 630 | 69.86 | 5.58 |
| *CaCRK16* | CA08g00770 | 535 | 58.83 | 9.37 |
| *CaCRK17* | CA08g00790 | 512 | 56.41 | 8.58 |
| *CaCRK18* | CA08g00810 | 674 | 74.81 | 8.47 |
| *CaCRK19* | CA08g00820 | 644 | 71.57 | 8.90 |
| *CaCRK20* | CA10g17720 | 1120 | 128.25 | 9.43 |
| *CaCRK21* | CA10g17730 | 682 | 75.75 | 6.29 |
| *CaCRK22* | CA11g08020 | 675 | 75.18 | 8.72 |
| *CaCRK23* | CA11g08030 | 649 | 71.89 | 8.86 |
| *CaCRK24* | CA11g16180 | 332 | 36.79 | 9.21 |
| *CaCRK25* | CA11g16200 | 408 | 45.16 | 8.69 |
| *CaCRK26* | CA11g16210 | 500 | 55.19 | 8.61 |
| *CaCRK27* | CA12g01330 | 639 | 70.38 | 8.74 |

* Genome accessions are from CM334 genome.
