# Supplementary material for: A cysteine-rich receptor-like protein kinase CaCKR5 modulates immune response against Ralstonia solanacearum infection in pepper
Source: BMC Plant Biol. 2021 Aug 19;21:382. doi: 10.1186/s12870-021-03150-y (PMC8375189; doi:10.1186/s12870-021-03150-y)
Supplement: Supplementary file 5 — Additional file 5. Original images for Fig. 4a, Fig. 5a and Fig. 6b. [file 12870_2021_3150_MOESM5_ESM.pdf]

Additional file 5

WT L3 L7

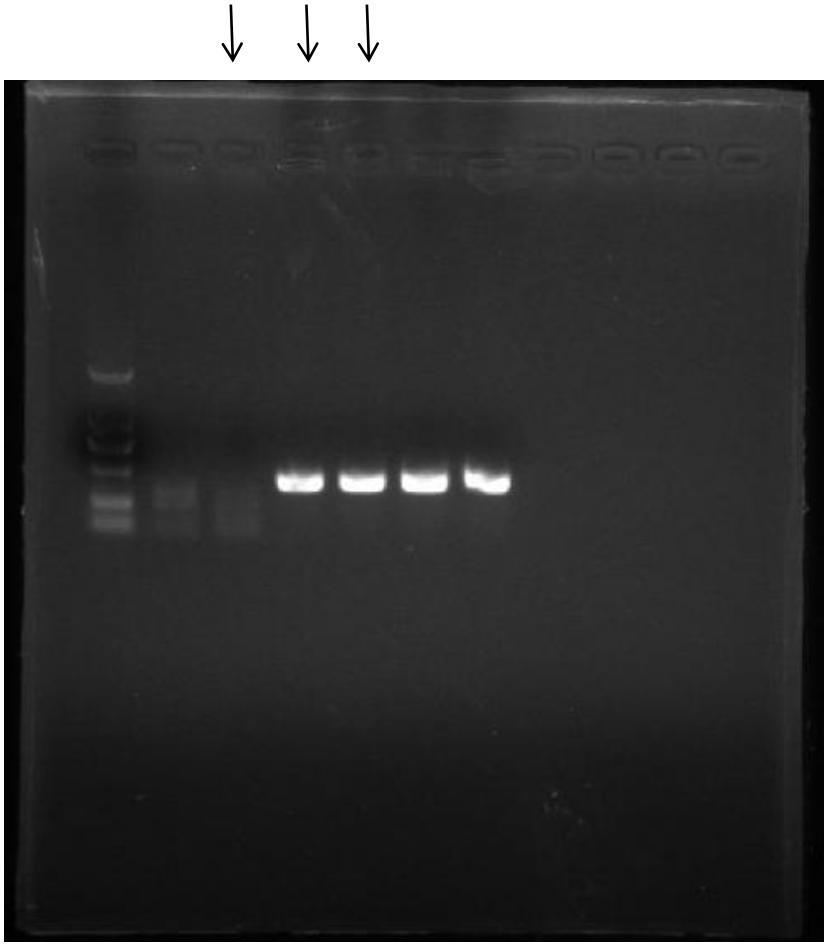

PCR for *CaCRK5*

WT L3 L7

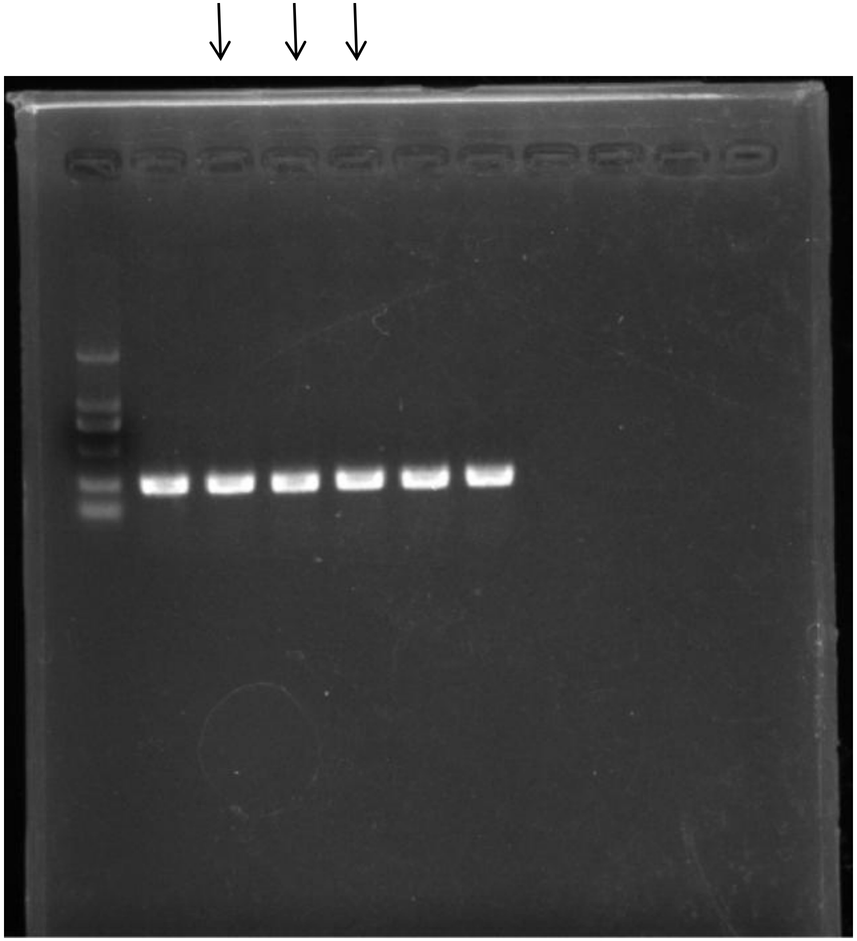

PCR for *NtActin*

Original image for Fig. 4a

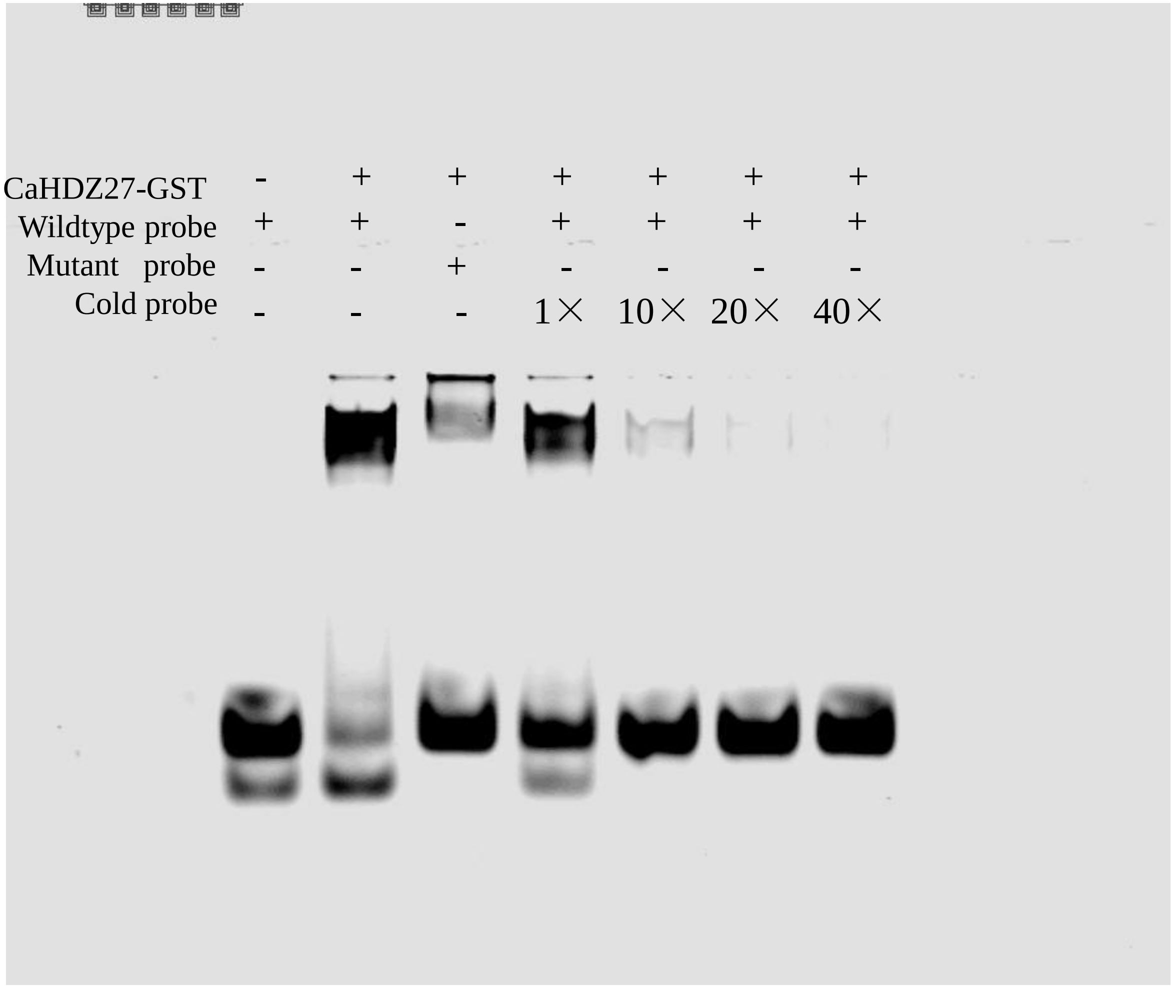

Original image for Fig. 5a

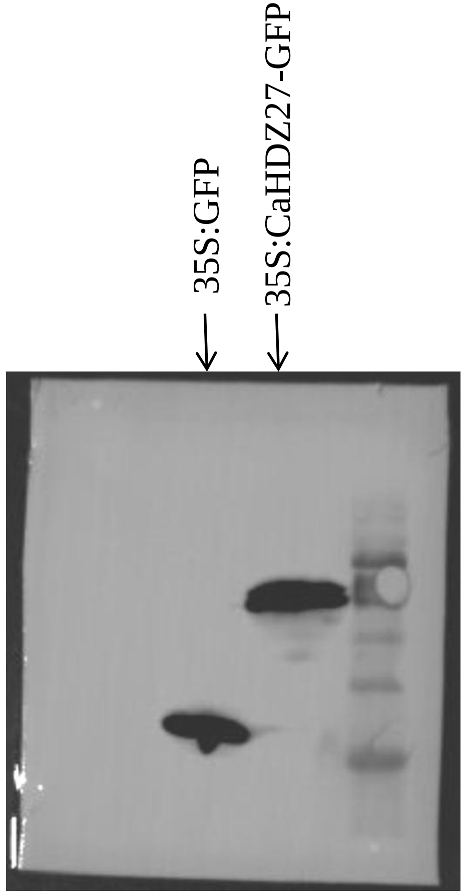

Western blotting

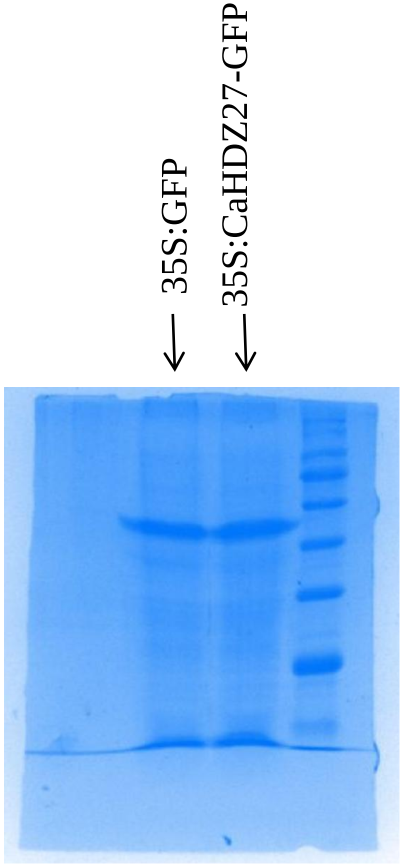

Coomassie brilliant blue

Original image for Fig. 6b
