# Supplementary material for: A cysteine-rich receptor-like protein kinase CaCKR5 modulates immune response against Ralstonia solanacearum infection in pepper
Source: BMC Plant Biol. 2021 Aug 19;21:382. doi: 10.1186/s12870-021-03150-y (PMC8375189; doi:10.1186/s12870-021-03150-y)
Supplement: Supplementary file 6 — Additional file 6. The information of realt time PCR primers. [file 12870_2021_3150_MOESM6_ESM.docx]

**Additional file 6.** The information of real time PCR primers

| **Genes** | **Assays** | **Primer sequences** | **Size**  **(bp)** | **Annealing temperatures** | **PCR**  **efficiency(%)** |
| --- | --- | --- | --- | --- | --- |
| *CaCKR5* | Real time PCR | F GGATTGCTCCGACTGCTTA  R TCCACTTTCTCCTCCTCAT | 295 | 69 | 97 |
|  | ChIP-qPCR | F TATAACCTTTTCTCCGAGTT | 185 | 65 | 95 |
|  |  | R TTAAGGGGCTATCGAGGC |  |  |  |
| *CaNPR1* | Real time PCR | F ACTTCTTCGCCGACGCCAAG | 190 | 72 | 101 |
|  |  | R GCCAACACATTCACCAGAGCATC |  |  |  |
| *CaDEF1* | Real time PCR | F GTGAGGAAGAAGTTTGAAAGAAAGTAC | 267 | 69 | 99 |
|  |  | R TGCACAGCACTATCATTGCATACAATTC |  |  |  |
| *CaACO1* | Real time PCR | F CCATTGTGGTCAACCTTGGC | 136 | 71 | 104 |
|  |  | R GCATCGCTTCCTGGATTGTAA |  |  |  |
| *CaSAR8.2* | Real time PCR | F CAGGGAGATGAATTCTGAGGC | 220 | 66 | 98 |
|  |  | R CATATGAACCTCTATGGATTTCTG |  |  |  |
| *CaActin* | Real time PCR | F AGGGATGGGTCAAAAGGATGC  R GAGACAACACCGCCTGAATAGC | 225 | 72 | 98 |
| *Ca18S rRNA* | Real time PCR | F CCGGTCCGCCTATGGTGTGCACCGGTCGTC  R GCAGTTGTTCGTCTTTCATAAATCCAAGAA | 285 | 72 | 97 |
| *NtPR2* | Real time PCR | F TGATGCCCTTTTGGATTCTATG | 175 | 69 | 96 |
|  |  | R AGTTCCTGCCCCGCTTT |  |  |  |
| *NtPR3* | Real time PCR | F CAGGAGGGTATTGCTTTGTTAGG | 222 | 71 | 103 |
|  |  | R CGTGGGAAGATGGCTTGTTGTC |  |  |  |
| *NtHSR201* | Real time PCR | F CAGCAGTCCTTTGGCGTTGTC | 173 | 72 | 101 |
|  |  | R GCTCAGTTTAGCCGCAGTTGTG |  |  |  |
| *NtHSR515* | Real time PCR | F TTGGGCAGAATAGATGGGTA | 499 | 69 | 97 |
|  |  | R TTTGGTGAAAGTCTTGGCTC |  |  |  |
| *NtActin* | Real time PCR | F TCACAGAAGCTCCTCCTAATCCA | 113 | 71 | 97 |
|  |  | R GAGGGAAAGAACAGCCTGAATG |  |  |  |
| *NtEF1α* | Real time PCR | F TGCTGCTGTAACAAGATGGATGC | 134 | 72 | 99 |
|  |  | R GAGATGGGGACAAAGGGGATT |  |  |  |
