# Supplementary material for: A cysteine-rich receptor-like protein kinase CaCKR5 modulates immune response against Ralstonia solanacearum infection in pepper
Source: BMC Plant Biol. 2021 Aug 19;21:382. doi: 10.1186/s12870-021-03150-y (PMC8375189; doi:10.1186/s12870-021-03150-y)
Supplement: Supplementary file 7 — Additional file 7. Melting curves in real time PCR. [file 12870_2021_3150_MOESM7_ESM.pdf]

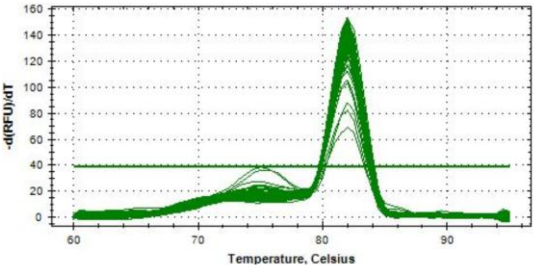

CaCKR5 real time PCR

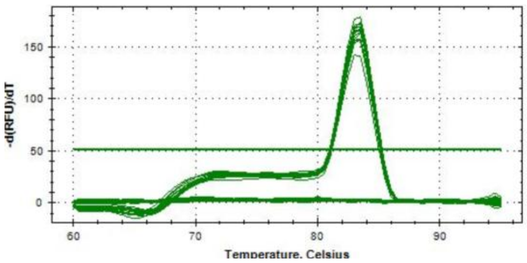

CaCKR5 ChIP-qPCR

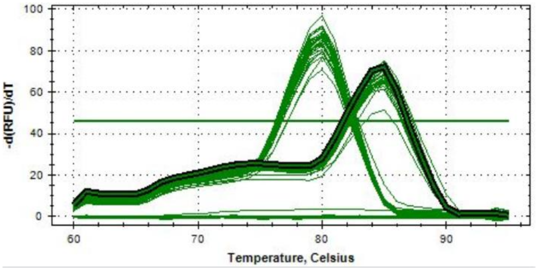

CaNPR1 and *CaDEF1*

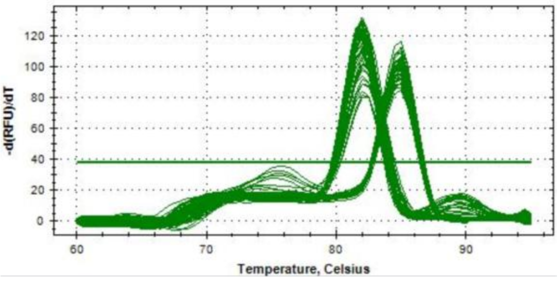

CaACO1 and *CaSAR8.2*

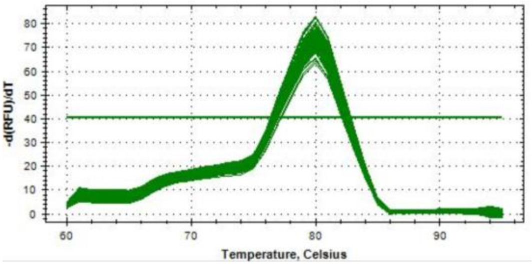

CaActin

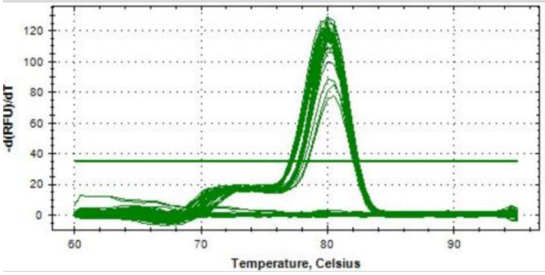

Ca18S rRNA

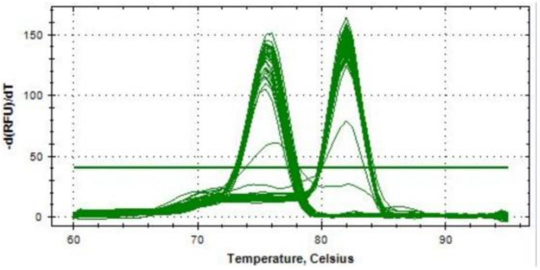

NtPR2 and *NtPR3*

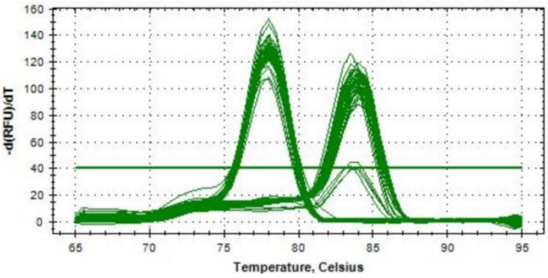

NtHSR201 and *NtHSR515*

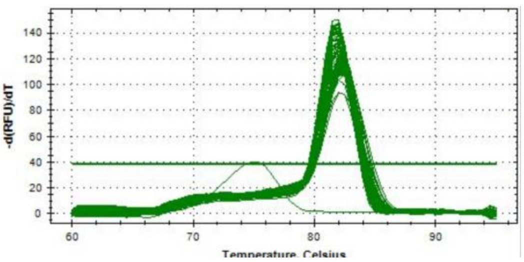

NtActin

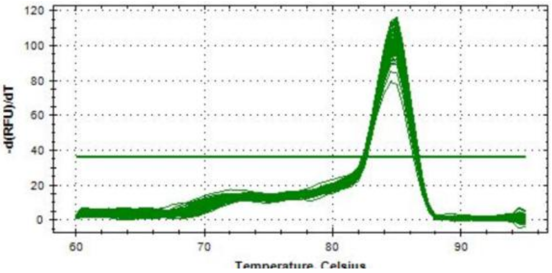

NtEF1α
